# Crystal Structure of the Human tRNA Guanine Transglycosylase Catalytic Subunit QTRT1

**DOI:** 10.3390/biom8030081

**Published:** 2018-08-24

**Authors:** Sven Johannsson, Piotr Neumann, Ralf Ficner

**Affiliations:** Department of Molecular Structural Biology, Institute of Microbiology and Genetics, GZMB, Georg-August-University Göttingen, 37077 Göttingen, Germany; sjohann1@gwdg.de (S.J.); pneuman2@gwdg.de (P.N.)

**Keywords:** TGT, QTRT1, queuine, tRNA modification, wobble base

## Abstract

RNA modifications have been implicated in diverse and important roles in all kingdoms of life with over 100 of them present on tRNAs. A prominent modification at the wobble base of four tRNAs is the 7-deaza-guanine derivative queuine which substitutes the guanine at position 34. This exchange is catalyzed by members of the enzyme class of tRNA guanine transglycosylases (TGTs). These enzymes incorporate guanine substituents into tRNA^Asp^, tRNA^Asn^ tRNA^His^, and tRNA^Tyr^ in all kingdoms of life. In contrast to the homodimeric bacterial TGT, the active eukaryotic TGT is a heterodimer in solution, comprised of a catalytic QTRT1 subunit and a noncatalytic QTRT2 subunit. Bacterial TGT enzymes, that incorporate a queuine precursor, have been identified or proposed as virulence factors for infections by pathogens in humans and therefore are valuable targets for drug design. To date no structure of a eukaryotic catalytic subunit is reported, and differences to its bacterial counterpart have to be deducted from sequence analysis and models. Here we report the first crystal structure of a eukaryotic QTRT1 subunit and compare it to known structures of the bacterial TGT and murine QTRT2. Furthermore, we were able to determine the crystal structure of QTRT1 in complex with the queuine substrate.

## 1. Introduction

RNA molecules have been shown to undergo extensive post-translational modification with 171 modifications known to date and over 100 of them occurring on transfer RNAs (tRNAs) [1,2]. In tRNA these modifications fulfill diverse functions, ranging from their influence on the three-dimensional (3D) structure to tuning of interactions with proteins. Incorporation of modifications into tRNA is also a dynamic process, as their distribution, as well as abundance level, might change upon nutrient alteration and stress changes [3]. The function of a particular modification is not only determined by its chemical properties but also highly dependent on its location in the tRNA molecule. While modifications in the structurally important D-loop and T-loop regions may alter and fine tune the tRNA’s three dimensional shape [4], modifications harbored in the anticodon stem loop are implicated to modify codon–anticodon interaction and/or are relevant during protein translation [5].

A prominent modification in the anticodon loop, which is present in the majority of bacterial and eukaryotic organisms, is the 7-deaza-guanine derivative queuine, which substitutes the nucleic base guanine at the wobble base position 34 of four tRNAs: tRNA^Asp^, tRNA^Asn^, tRNA^His^, and tRNA^Tyr^ [6]. In contrast to eukaryotes, bacteria synthesize queuine de novo. Starting from guanosine 5′-triphosphate the queuine precursor 7-(aminomethyl)-7-deazaguanine (preQ1) is built up outside the tRNA [7,8,9,10,11,12,13]. The incorporation of preQ_1_ into the tRNA is catalyzed by the enzyme tRNA-guanine transglycosylase (TGT) in a proposed ping-pong mechanism [14]. The 3D structure of a eubacterial TGT was firstly described by Romier et al. for the *Zymomonas mobilis* enzyme, a 43 kDa protein. This eubacterial TGT (bacTGT) harbors an eight stranded beta (β/α)_8_ triose-phosphate-isomerase like fold, a (β/α)_8_ TIM barrel, which is a characteristic of this enzyme class that provides the structural basis for substrate binding and catalysis [15]. Furthermore, it contains a zinc ion binding site which, in concert with the TIM barrel, provides the scaffold for binding of the tRNA substrate by this enzyme class [16,17,18].

Due to the scarce structural knowledge, insights into tRNA recognition are mainly provided by the structure of the archeosine *Pyrococcus horikoshii* TGT (arcTGT) in complex with a full length tRNA. However, arcTGT specifically targets the G15 base of cognate tRNAs for modification, rather than G34 like the eubacterial and eukaryotic enzymes [18,19,20]. Insights into the tRNA protein interaction and the underlying mechanism of the transglycosylation reaction for the bacterial enzyme were provided by the crystal structure of the covalently bound tRNA anticodon stem loop to bacTGT from *Z. mobilis*. Interestingly, this complex structure revealed Asp280 to execute the nucleophilic attack on the C atom of the *N*-glycosidic bond between the ribose and the guanine base rather than Asp102, the residue that was originally proposed to be involved in this cleavage [15,21]. The consequence of this nucleophilic attack is the formation of a covalent tRNA-enzyme reaction intermediate in which RNA and protein are linked by a covalent bond between the Asp280 and the remaining ribose of the tRNA backbone [15]. The cleaved guanine base is free to dissociate from the active site and is subsequently replaced by the preQ1 substrate. Formation of the *N*-glycosidic linkage between the tRNA and preQ1 is then achieved by deprotonation of the preQ1’s N5 atom through the TGT’s Asp102, which facilitates a nucleophilic attack of preQ1 on the covalent tRNA Asp280 intermediate leading to formation of the final reaction product. Finally, the modified tRNA product dissociates from the enzyme in a rate limiting step [22].

This mechanism of queuine incorporation by bacterial TGTs has recently become of interest for drug design as TGT has been identified as an important virulence factor. In *Shigella*, a gram negative bacterium and the causative factor of shigellosis in human, the absence of a functional TGT strongly reduces pathogenicity, allegedly caused by queuine hypomodification of the mRNA encoding the transcriptional factor virF [23,24,25]. Therefore the bacterial TGT has emerged as a valuable target for structure based inhibitor design [26,27]. Beside infection by *Shigella*, queuine has also been proposed to promote host infection of the pathogenic ameba *Entamoeba histolytica*, which might scavenge the queuine base from gut bacteria [28].

As for *E. histolytica,* most eukaryotes possess TGT enzymes, but, in contrast to their bacterial counterparts, they do not encode for enzymes necessary to build up queuine and therefore have to incorporate the queuine base, instead of a precursor, into RNA targets. The essence of queuine in eukaryotes requires humans and other organisms to scavenge this modified base from nutritional sources and/or from microbiomes [29,30]. Likely to meet these altered demand and more complex pathways and regulation in eukaryotic cells, properties of eukaryotic TGTs have emerged to show significant differences to their bacterial counterparts. In contrast to the homodimeric architecture of the bacterial and archeal TGT, the eukaryotic, heterodimeric TGT is composed of a catalytically active queuine tRNA-ribosyltransferase subunit 1 (QTRT1) and a catalytically inactive QTRT2 subunit. This quaternary structure has been confirmed to be essential for catalyzing the incorporation of queuine into tRNA [31]. Recently, the crystal structure of the noncatalytic mouse QTRT2 subunit has been solved and found to confirm the prior suggestion that this subunit is incapable to catalyze the guanine to queuine exchange reaction as catalytic residues, and the residues involved in substrate coordination are largely replaced by chemically different amino acids and thus are extremely unlikely to facilitate queuine binding or even the transfer reaction [32]. In contrast, alignment of the human QTRT1 sequence with the *Z. mobilis* TGT reveals this subunit to be likely catalytically active with relevant residues in place. However, despite the potential importance of this subunit as a drug target, its structure remains unknown.

Understanding the mechanism of queuine incorporation by the eukaryotic TGT enzyme is of great interest as altered presence of queuine in TGT substrates has been associated with various disease related effects such as cancer. Here queuine hypomodification in tRNA has been linked to increased cancer progression [33,34] as well as impaired cell differentiation [35]. Furthermore, mice which were genetically modified to lack a functional TGT enzyme showed symptoms similar to those associated with the disease polyphenylketonurea as they were deficient in producing tyrosine from phenylalanine [36]. The underlying mechanism of this effect is not known to date, but Rakovich et al. were able to link this observation to absence of QTRT1 and consequently to the lack of queuine in the tRNA pool.

Additionally, queuine has been proposed to increase stress tolerance [37], an effect unraveled more than 20 years ago of which the mechanistic details were only recently described. Here, queuine modification of tRNA^Asp^ has been proven in vivo and in vitro to trigger activity of the methyltransferase Dnmt2 which results in establishment of m^5^C modification at cytosine 38 (m^5^C38) of this tRNA [38,39].

Despite the numerous implications of TGTs in diseases in mammals, little is known on the structural level of queuine incorporation by eukaryotic TGT enzymes, as information about the catalyzed reaction was deducted from analysis of bacterial TGT structures through homology modeling. Recently, the QTRT2 crystal structure allowed the first structural insights into the arrangement of the noncatalytic subunit and potential dimer assembly, leaving the biological significance without a functional catalytic site unsolved. The 3D structure of a eukaryotic TGT catalytic subunit, which would provide reliable information about the architecture of the active site and thus the mechanism, is not known.

Here we report the crystal structure of the human QTRT1 subunit in its apo form and in complex with the queuine base. The performed analysis reveals the QTRT1 fold to be highly related to the bacterial TGT of *Z. mobilis* while the active site is altered in order to accommodate queuine base, which is bulkier than preQ1. Furthermore, comparison with the QTRT2 crystal structure provides evidence that this noncatalytic subunit is unlikely to harbor the ability of binding queuine in the active center.

## 2. Materials and Methods

### 2.1. Cloning of Human TGT

QTRT1 and QTRT2 were ordered as synthetic genes from Invitrogen GeneArt (ThermoFisher Scientific, Waltham, MA, USA) as codon optimized sequences for expression in *E. coli*. QTRT2 and QTRT1 were cloned sequentially into the pCDF Duett 1 vector using the restriction enzymes NdeI/KpnI and EcoRI/HindIII, respectively, with QTRT1 harboring a N-terminal PreScission site for proteolytic cleavage of the 6xHis tag.

### 2.2. Expression and Purification

The human TGT heterodimer QTRT1 and QTRT2 were co-expressed from pCDF-Duett vector with a cleavable N-terminal 6xHis tag to QTRT1 (his-hTGT) in *Escherichia coli* BL21(DE3)-STAR cells using autoinduction. The medium was supplemented with additional 100 µM ZnCl_2_. Cells were grown at 18 °C for 50 h before harvesting. Cell pellets were flash-frozen in liquid N_2_ and stored at −80 °C until further use. For purification purposes the cells were thawed and disrupted by microfluidization (M-110S Microfluidizer (Microfluidics, Westwood, MA, USA)) (50 mM HEPES pH 7.5, 100 mM NaCl, 10 mM imidazole). Soluble protein was isolated by ultracentrifugation at 50,000 g for 30 min. His-hTGT was loaded onto His-Talon Superflow (GE Healthcare, Chicago, IL, USA) and washed with additional 1 M LiCl. Protein was eluted in a 20 CV gradient to 50 mM HEPES pH7.5, 100 mM NaCl, 500 mM imidazole. Target protein containing fractions were pooled and supplemented with PreScission protease (1:100 *w/w*) and incubated for 16–18 h under mild agitation. The protein was further purified by Superdex S200 (GE Healthcare) size exclusion chromatography (20 mM HEPES pH 7.5, 100 mM NaCl). hTGT was concentrated to 6–8 mg/mL and further processed, or flash frozen in liquid N_2_ and stored at −80 °C until further use.

### 2.3. Crystallization

QTRT1 was crystallized using sitting-drop or hanging drop vapor diffusion technique with protein concentration adjusted to 6 mg/mL as determined with Bradford reagent (Bio-Rad, Hercules, CA, USA). Equal volumes of protein containing solution and crystallization condition (100 mM Tris pH 7.8, 200 mM KBr, 200 mM KSCN, 3% (*w/v*) γ-polyglutamic acid-LM (PGA-LM), 5% PEG 4000) were mixed. Crystallization was performed in EasyXtal-15-well tool plates (Qiagen, Hilden, Germany) with the cap loosened at a ¼ turn to allow for evaporation or in loosely sealed 24 well Cryschem plates (Hampton Research, Aliso Viejo, CA, USA). Crystals were obtained after five to seven days at 20 °C from crystallization drops of 0.5–6 µL total volume. To obtain QTRT1 in complex with queuine, respective crystals were soaked in the crystallization condition containing the free queuine base at 50 µM concentration. Crystals were cryo-protected by a stepwise increase of glycerol and PEG400 concentration to 15% (*v/v*) each.

### 2.4. Data Collection, Molecular Replacement and Refinement

X-ray data collection was performed at −173 °C; diffraction images were collected at P14 beamline, operated by EMBL Hamburg, at the PETRA III storage ring (DESY, Hamburg, Germany). Diffraction images were indexed, integrated, and scaled using the XDS-package [40]. The structure of QTRT1 was solved by molecular replacement by PHASER [41], as implemented in the CCP4 suite [42]. The crystal structure of the *Zymomonas mobilis* tRNA guanine transglycosylase [16] (PBD-ID: 1PUD) was used as a model and modified with CHAINSAW, as implemented in the CCP4 suite. MR-SAD was performed using the phenix.autosol pipeline. To overcome the difficulties in manual model rebuilding, both QTRT1 monomers were subjected to energy and density-guided refinement in Rosetta [43] utilizing constrains from the MR-SAD electron density map. Model building was done with subsequent iterative cycles of automated refinement with PHENIX [44] and manual model adjustments in Coot [45]. Crystallographic values are listed in the Appendix A.

### 2.5. Q-Base Synthesis

The queuine base was synthesized as described previously, starting from inexpensive methyl α-d-galactopyranoside for the (1R,2S,3S)-1-bromo-2,3-*O*-isopropylidene-cyclopent-4-ene building block and preQ1 [38].

## 3. Results

To obtain the structure of the human TGT, we cloned the coding sequences for both human TGT subunits, QTRT1 and QTRT2, into a pCDF Duett vector and co-expressed both full length constructs in *E. coli* BL21 DE3 cells. The heterodimer was purified with subsequent affinity purification, cleavage of the 6xHis tag from the QTRT1 subunit and size exclusion chromatography, yielding a pure, nucleotide free, and active TGT heterodimer. The purified TGT heterodimer was submitted to crystallization process immediately after purification. The protein containing solution was mixed with the crystallization condition (100 mM Tris pH 7.8, 200 mM KBr, 200 mM KSCN, 3% (*w/v*) PGA-LM and 5% PEG 4000) in a 1:1 ratio. Crystals were obtained after 5 to 14 days as plates with varying sizes of 40 to 150 µm utilizing sitting and hanging drop experimental setups. Crystallization was favored by slowly increasing the concentration of components in the drop with controlled evaporation. The crystals were cryoprotected and exposed to synchrotron radiation. Multiple diffraction images datasets of the best diffracting crystal were collected, processed with XDS and subsequently merged with a resolution cutoff at 2.45 Å. The space group was identified to be C2. We were able to obtain initial phases by molecular replacement using the crystal structure of the *Z. mobilis* TGT monomer (Protein Data Bank (PDB)-ID: 1PUD) [16]. The crystal’s asymmetric unit comprises two molecules with only one of them (chain A) exhibiting reasonable fit to electron density after initial refinement steps. The other molecule (chain B) only marginally fit the electron density and no interpretable density was observed for about 160 of the N-terminal amino acids. For validation of the molecular replacement (MR) model we used the initial phases from the MR and performed MR-SAD utilizing the weak anomalous signal of the merged dataset following by model rebuilding with Rosetta software. MR-SAD confirmed location of the zinc ion in both molecules occupying the asymmetric unit and improved phases which allowed to build both molecules simultaneously, thus advancing productivity during the model building and refinement process.

The purified TGT used for protein crystallization consisted of both subunits as confirmed by SDS-PAGE and was catalytically active, as shown by Q-incorporation into G34tRNA^Asp^ [38]. However, structural analysis of the asymmetric unit’s content revealed both molecules to be the catalytic QTRT1 subunit.

**Figure 1:**
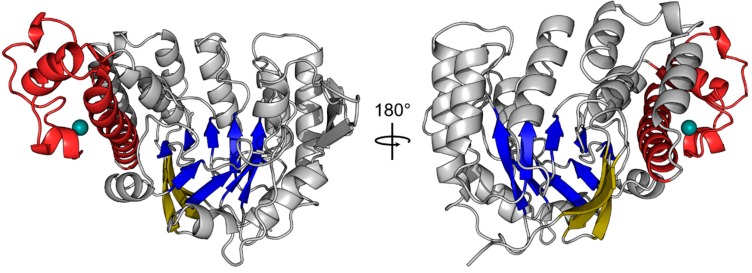
Crystal structure of the human tRNA guanine transglycosylase (TGT) catalytic subunit queuine tRNA-ribosyltransferase (QTRT1). The crystal structure of the QTRT1 monomer (PDB-ID: 6H42) is presented as cartoon. The eight β-sheets of the (α/β)_8_ barrel at the core of the protein are shown in blue with a three stranded β-sheet acting as a lid (yellow) which covers the bottom of the barrel. QTRT1 additionally harbors a zinc binding domain (red), which tetragonally coordinates a zinc-ion (turquoise). Both structures show the same molecule rotated 180° around the y-axis.

The structure consisting of two QTRT1 subunits (PDB-ID: 6H42) was refined to a final R_work_ and R_free_ of 20.13 and 23.96% respectively, with the first subunit (chain A) exhibiting B-factors in the mid 60 Å^2^ while chain B appeared to be more flexible within the crystal lattice with an averaged B-factor of around 70 Å^2^. However, analysis of both molecules with respect to folding revealed no significant discrepancies between them. Analysis of the human QTRT1 catalytic subunit reveals the enzyme to possess a bended (α/β) barrel, consisting of an eight stranded β-barrel at the core and eight flanking helices (Figure 1). In addition, the protein contains a Zn^2+^ binding domain, in which the zinc ion is coordinated by a triangular arrangement of three cysteine residues Cys317, Cys319, and Cys322, while the α-helix bound His248 completes the tetragonal coordination of the metal ion. A detailed listing of data processing and refinement statistics can be found in the crystallographic table (Appendix A). Furthermore, an alignment of the sequences from the human QTRT1, *Z. mobilis* TGT and mouse QTRT2 is presented in Appendix B.

**Figure 2:**
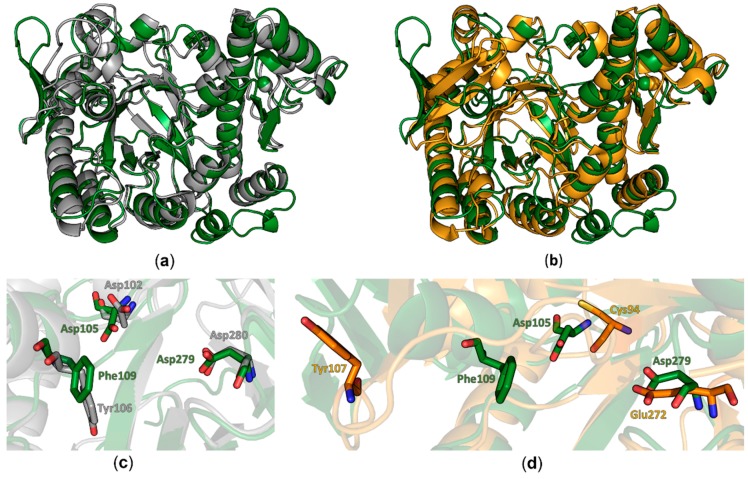
Structural comparison of QTRT1 to bacTGT and QTRT2. (**a**) Overlay of the human QTRT1 structure (green) with the bacterial TGT monomer of *Zymomonas mobilis* in grey (PDB-ID: 1PUD). Both structures are depicted as cartoons. The zinc ion is presented as a sphere in the respective color of the structure. (**b**) Alignment of the human QTRT1 structure (green) to the monomer structure of mouse TGT noncatalytic subunit QTRT2 (orange). Both structures are presented as cartoons with the respective zinc ions depicted as spheres. (**c**) Zoom into the active site of the overlay shown in (a) with according coloring of the molecules. bacTGT active residues Asp102 and Asp280 as well as the preQ1 stacking Tyr106 are depicted as grey sticks. Equivalent residues in QTRT1 are presented as green sticks. (**d**) Zoomed in view into the QTRT1 active site. QTRT1 residues presented in (c) are depicted in green. The respective residues in QTRT2 (orange) were identified upon alignment of the two protein sequences. The QTRT1 Phe109 aligned QTRT2 residue Tyr107 is displaced in the QTRT2 structure.

Comparison of the human QTRT1 structure with the previously reported structure of its bacterial counterpart from *Z. mobilis* reveals both proteins to share a fairly similar fold with a root-mean-square deviation (RMSD) of 2.86 Å (calculated between all equivalent Cα positions). Especially the arrangement of the (α/β)_8_ barrel and the zinc ion binding domain in this protein seems to be structurally conserved from bacteria to eukaryotes. Differences between both structures were observed in the conformation of the region comprising amino acids 114–138 which adopts a three stranded beta sheet with an extended loop between the β-strands two and three that folds into a more open conformation compared to the bacterial TGT. Furthermore, we did observe electron density for an additional C-terminal α-helix. Most significant differences are observed in regions lacking a defined secondary structure. In the bacTGT enzyme two aspartate residues have been identified as catalytically active, with Asp280 performing the initial nucleophilic attack and the nucleophilic Asp102 sidechain supporting the formation of the reaction product. In the reported QTRT1 structure we observed conserved Asp105 and Asp279, occupying structurally equivalent positions, thus arguing for a conserved catalytic mechanism (Figure 2). As a consequence of the purification strategy, which included a high salt concentration, we did not observe additional electron density in the active site that may correspond to a bound ligand.

Comparison of the reported structure of the human QTR1 with the crystal structure of the noncatalytic QTRT2 subunit from mouse unveiled more striking differences. While the beta barrel and the zinc ion binding domain colocalize well in an alignment of both monomers (Figure 2b), more substantial differences could be observed in the active core. The catalytically active Asp279 of QTRT1 is exchanged by a chemically similar but more spacious glutamate in QTRT2 (Glu272) and Asp105 of QTRT1 is replaced by a chemically distinct cysteine residue (Cys94). The Phe109 residue, which is structurally equivalent to the bacTGT Tyr106 in QTRT1 (Figure1b), is replaced by a tyrosine in QTR2 (Tyr107). As a consequence of an altered conformation of the underlying polypeptide chain, Tyr107 is displaced from the active site in QTRT2. Comparison of the residues flanking the active site reveals striking differences between QTRT2 and both QTRT1 and bacTGT. In QTRT1 amino acids 108 to 114 adopt a α-helical conformation that consecutively results in placement of Phe109 in close proximity to the active aspartate residues, an arrangement highly similar to the respective residues in bacTGT. This α-helix is likely not to obstruct substrate binding as the active site is still accessible for substrates. Therefore, we refer to this structural arrangement as the “competent” conformation. In contrast, the respective stretch of amino acids in QTRT2 adopts a strikingly different conformation. Instead of formation of the mentioned helix, the peptide chain protrudes into the degenerated active site as a loop. This conformation is stabilized by stacking formed between the Try116 and Pro104 side chains and results in the mentioned displacement of Tyr107. We refer to this arrangement as the “incompetent” conformation, as is very likely would interfere with substrate binding.

Binding of the bacterial TGT to its substrate preQ1 has previously been proposed as a valuable target for drug design [46]. A dilemma for structure based drug development has been the unknown structural differences of the bacterial and eukaryotic TGT active site, as avoiding cross-targeting of the human TGT is one of the main challenges in small molecule drug development. Efforts with structural prediction, alignments and even engineering of the bacterial TGT’s active site to mimic its eukaryotic counterpart through amino acid exchanges have been undertaken to tackle this problem, but are still not the ultimate proof [47].

**Figure 3:**
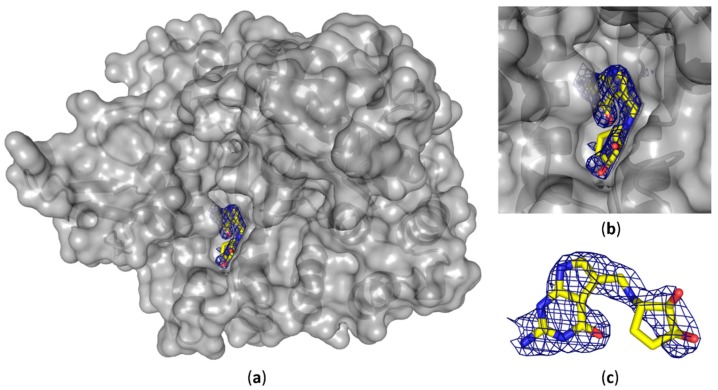
Crystal structure of human QTRT1 in complex with queuine. (**a**) Crystal structure of the human QTRT1 subunit in complex with queuine (PDB-ID: 6H45). QTRT1 is depicted in grey with a partly transparent surface and underlying polypeptide chain presented as a cartoon. Queuine is presented in yellow as sticks with blue nitrogen atoms and oxygens in red. DF_0_-mF_c_ map for the omitted queuine base is shown as blue mesh with 2.0 σ contour level. (**b**) Close-up view into the active site with the complexed queuine base. (**c**) Conformation of the queuine base bound to QTRT1. Blue electron density shows DF_0_-mF_c_ omit map contoured at 2.0 σ.

We were able to solve the crystal structure of the QTRT1 subunit in complex with queuine (PDB-ID: 6H45), by soaking the fully modified base into QTRT1 crystals. As a consequence of soaking, we did observe additional electron density corresponding to queuine in the active site (Figure 3), that clearly is absent in the apo QTRT1 structure. Binding of the queuine 7-deaza-purine moiety is promoted by stacking interactions and the shape of this density corresponds well to the queuine chemical structure. In addition, the excess electron density map localizes at the site which is equivalent to the preQ1 binding site in the bacterial TGT enzyme. The 7-deaza-guanine moiety is stacking between Phe109 and Met 259 side chains which have previously been implicated in queuine binding in eukaryotic TGTs. Furthermore, correct positioning of the queuine base for the enzymatic reaction is largely provided through polar contacts formed with Asp159. The exocyclic oxygen forms contacts with the polypeptide mainchain at residue Gly229, thus completing the coordination of the 7-deaza-purine. We also did observe density in the mF_o_-DF_c_ omit map for the additional cyclopentene-*cis*-diol, a hallmark of this modification, which distinguishes queuine from the bacTGT substrate preQ_1_. The di-hydroxy-cyclopentene moiety is located in a grove of the protein surface, distant from the QTRT1 catalytic residues and thus also to the anticipated binding site of the RNA substrate. While the space to accommodate the cyclopentene ring is provided by the Gly232 and Gly233 residues, its coordination is achieved by polar contacts of the cyclopentene C5 hydroxy group and Ser164 of the protein chain. Compared to the QTRT1 apo structure the conformational changes induced upon queuine binding are rather minimal. The QTRT1 main chain and side chains involved in stacking or polar contacts formation with queuine exhibit a fairly similar conformation in both, the complex and the apo structure. The most striking difference is observed for Ser231 which is shifted away from the queuine ligand presumably to provide necessary space for accommodation of the cyclopentene moiety.

## 4. Discussion

To date structural investigation of tRNA guanine transglycosylases mainly focused on the bacterial TGT of *Z. mobilis* and the archeal TGT from *Pyrococcus horikoshi*. While these enzymes catalyze an incorporation of the modified bases preQ1 at position 34 and preQ0 at position 15 into cognate tRNAs respectively, the structure of the eukaryotic TGT that incorporates the finally modified queuine base was unknown. Here we report the first crystal structure of the human QTRT1, which is the catalytic subunit of a eukaryotic TGT enzyme.

The QTRT1 crystal structure resembles the previously reported two hallmarks of TGTs, namely the (α/β)_8_ barrel that builds the supporting structure for the catalytic center, and a zinc binding domain. We find these two structural motifs to be organized in a fairly similar manner as was observed in the previously reported structures of the eubacterial *Z. mobilis* TGT and the noncatalytic mouse QTRT2 protein.

Furthermore, we were able to obtain the structure of human QTRT1 in complex with queuine. Presence of the bound substrate has been confirmed by the calculated mF_0_-DF_c_ map (Figure 3). Queuine localizes at the proposed binding site which corresponds to the preQ1 binding site in bacTGT. We tried also to obtain crystals with queuine present in the crystallization condition, but were unsuccessful to obtain crystals of suitable quality. Therefore, we chose to soak the ligand into apo crystals. We like to note that soaking vs. co-crystallization has the disadvantage that conformational changes upon ligand binding might not be accounted for, which might result in ligand positional deviations between the soaked and the co-crystallized structure. However, we find the queuine base in our complex structure is consistent with results from a previous study by Biela et al., that aimed to enable the bacTGT enzyme to bind the queuine base [47]. The authors identified residues that may allow for binding of queuine in the human QTRT1 by homology modeling. They were able to observe electron density for the queuine’s dicyclic purine when they introduced Tyr106Phe and Cys158Val mutations in bacTGT to mimic the queuine binding site of the human TGT. However, they were not able to locate the cyclopentene moiety. Our QTRT1 queine complex structure confirms their observation that the human QTRT1 Phe109 (the human equivalent of Try106 in bacTGT) stabilizes queuine binding through stacking with the purine part of the molecule. We can also confirm that indeed the bacTGT’s Cys158 residue colocalizes with Val161 of the human QTRT1, when both structures are aligned. Furthermore, we observe that absence of a sidechain at position 232 and 233 in QTRT1 does allow the accommodation of the bulky cyclopentene-diol ring which would otherwise be obstructed. This is not the case in the bacTGT enzyme, where instead of Gly232, a valine (Val233) is present at this location, that would interfere with binding of the cyclopentene.

**Figure 4:**
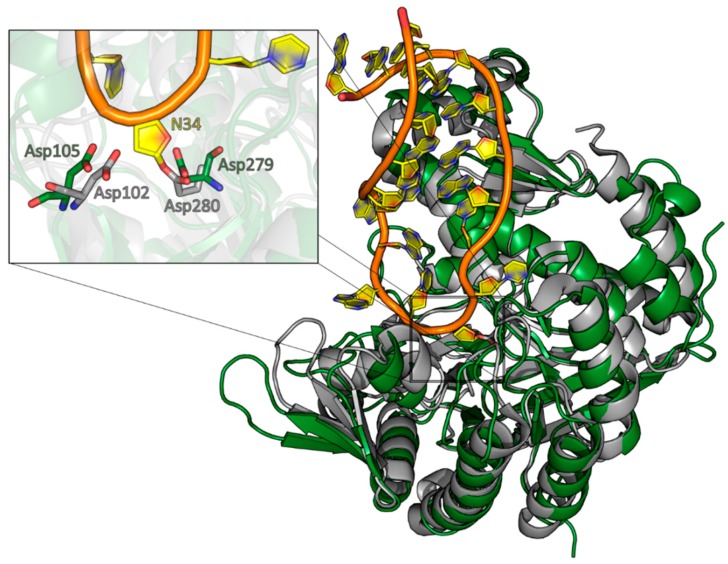
Alignment of the QTRT1 crystal structure with the *Z. mobilis* TGT RNA complex. The crystal structure of QTRT1 (green) was aligned to the protein part of the *Z. mobilis* TGT structure in complex with a tRNA anticodon stem loop (PDB-ID: 1Q2R). The complex structure was stalled by addition of 9-de-azaguanine thus exhibiting the deglycosylated state of the guanine 34. The zoomed view into the active site (**left**) shows the covalent link of the N34 ribose to the catalytic Asp280 residue and the close by Asp102, which is suggested to perform the nucleophilic attack on preQ1 in the bacterial enzyme. The overlay with QTRT1 finds the proposed catalytic residues D279 and D105 at the respective sites.

A structural alignment of the QTRT1 structure with the structure of the bacTGT solved in complex with an RNA substrate reveals Asp105 and Asp279 occupying the same positions as the respective catalytic residues in the catalytic bacTGT enzyme (Figure 4). The observation that queuine is bound by QTRT1 at the respective site where preQ1 is bound in the bacTGT enzyme argues for a conserved catalytic mechanism, although the substrate base is different.

While we do observe the structure of QTRT1 to be closely related to the structure of the bacTGT, its atomic model exhibits substantial differences to the previously described structure of the noncatalytic QTRT2 subunit. Previously reported results indicate that this subunit does not exhibit transglycosylation activity due to mutagenesis of active site residues [31]. In particular, we find the QTRT1 Asp279 residue to be replaced by a glutamate in QTRT2, that chemically harbors the potential to carry out a nucleophilic attack, however a potential reaction intermediate could not be released, as for this step the necessary Asp105 is replaced by a cysteine in QTRT2. Furthermore, we find the queuine binding site to be degenerated in QTRT2. While in QTRT1 queuine is coordinated by stacking between Phe109 and Met259 we do observe dislocation of the respective QTRT2 Tyr107 residue and a mutation of the methionine to cysteine (Cys252). As a result, high affinity binding of queuine to the active site of QTRT2 is unlikely. Compared to QTRT2, QTRT1 harbors all catalytic residues and is capable of binding the modified base. However previously reported biochemical assays reported that QTRT1 alone does not facilitate the transglycosylation reaction. From our perspective the heterodimerization with QTRT2 might provide an additional binding scaffold for the tRNA substrate that could be essential for TGT activity, however this needs to be proven experimentally.

**Figure 5:**
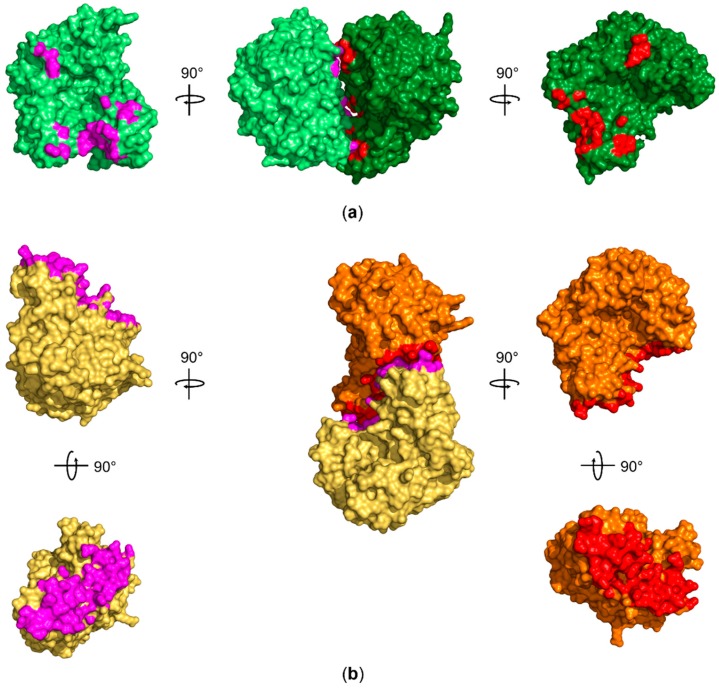
Comparison of the approximately 720 Å^2^ interface area observed for human QTRT1 with the interface area of the bacterial TGT homodimer. (**a**) Both QTRT1 molecules A and B are shown in dark green and pale green, respectively. Residues within 5 Å distance of the other molecule are shown in red for molecule A and in magenta for molecule B. (**b**) The interface area between the *Z. mobilis* TGT (PDB-ID: 1PUD) homodimer subunits, that are depicted in orange and pale orange, extends to approximately 1600 Å^2^. The orange subunit was aligned to QTRT1 molecule A (dark green) to outline the different interaction site compared to QTRT1. Residues within 5 Å of the respective other subunit are again presented in red and magenta. All structures in (a) and (b) are depicted as surface representations.

Furthermore, we asked why we do observe only QTRT1 in our crystals, although the crystallization drop contained the purified and catalytically active heterodimer. Prior studies of the mouse TGT quaternary structure by Behrens et al., employing mass spectrometry under nondenaturing conditions, evaluated the main fraction of an equimolar mixture of QTRT1 and QTRT2 to form the heterodimeric arrangement of the two subunits. However, the homodimeric subunit arrangements of both QTRT1 and QTRT2 have been observed previously. Interestingly, the vast majority of the QTRT2 in absence of the catalytic subunit formed homodimers. While the biological consequence of this observation remains elusive, the authors were also able to observe a homodimeric arrangement of this subunit in the crystal structure. The same investigation of the catalytic QTRT1 subunit revealed the ability of the catalytic subunit to form stable homodimers, however to a much less extend with a fraction of only 20% of proteins being self-associated in solution. Analysis of the QTRT1 crystallization condition revealed the presence of highly chaotropic thiocyanate, which might promote destabilization of the TGT heterodimer in the crystallization droplet. Furthermore, we do observe a PGA molecule at the interface of the asymmetric units promoting contact of the B-chain with a symmetry mate through stacking between symmetry equivalent Arg184 residues. The interface is further extended by a bromide ion, which is also a component of the crystallization condition. We further evaluated the quaternary structure of both proteins in the asymmetric unit and found the interaction not to be promoted by presence of components of the crystallization condition. However, although the active site is exposed to solvent, binding of a bulky substrate tRNA to one or the other catalytic site is most likely sterically hindered by presence of other molecules in the crystal lattice. We calculated the interaction interface to be of 719.9 Å^2^ with the free dimerization enthalpy of ΔG = −9.8 kcal/mol which does not suggest that this dimer is stable in solution [48]. Investigation of potential biological assemblies that include one component of the asymmetric unit and a contacting symmetry mate also did not find a stable dimer nor an arrangement that exhibited similar coordination of both subunits comparably to the one observed for the *Z. mobilis* homodimer or the proposed QTRT2 biological assembly (Figure 5).

Therefore, our crystal structure supports previous observations that QTRT1 is mainly monomeric in solution, and the dimer architecture we observe in the asymmetric unit is likely caused by crystal packing. The structures of QTRT1 and QTRT2 both support previous observations that a fraction of the eukaryotic TGT heterodimer is dissociated in solution however further biochemical studies are necessary to identify its biological significance.

The eukaryotic TGT has been proven to incorporate the fully modified queuine base, however, the introduction of queuine into the tRNA might not represent the final step for all queuine molecules after incorporation. Queuine has been observed to undergo further modification by *O*-mannosylation at the C4 hydroxy group of the cyclopentene diol moiety in humans and rats [49,50]. The question arises if the free queuine base is glycosylated or this hypermodification is established after introduction into the tRNA by TGT. While in humans only the O-linkage of mannose was observed, *O*-mannose or *O*-galactose was found to occur on queuine in samples derived from rat liver [50]. Biochemical investigation of the isolated, endogenous glycosyltransferase from rat liver found the enzyme to be specific for queuine in the context of tRNA^Asp^ as no mannosylation was observed for Q34tRNA^Asn^ and Q34tRNA^His^ while tRNA^Tyr^ was found to harbor galactose modification exclusively [51]. Therefore, the target seems to be defined not only by queuine chemical structure but also by tRNA structural properties. In our crystal structure of the queuine QTRT1 complex, we do observe the queuine cyclopentene C4 OH group to be exposed to the solvent. Presence of an additional sugar at this position would be possible and likely not interfere with coordination of the queuine base by the identified residues. Therefore, our QTRT1 crystal structure renders binding of a glycosylated queuine base into the active site possible in absence of a tRNA substrate. However, whether this would also be the case with a bound tRNA molecule still needs to be structurally investigated.

## 5. Conclusions

The herein reported structure of the human QTRT1 provides the first structural insights into the structural arrangement of a eukaryotic TGT active subunit and its active site. While previous structural investigations had to rely on homology modeling and engineering of the bacterial TGT, the herein reported crystal structures provide, for the first time, direct structural data about the location of catalytic residues in the active site and how the queuine substrate is bound. Furthermore, our results provide strong evidence for the previously deducted conservation of the TGT catalytic mechanism from eubacteria to eukaryotes.

## Appendix A

**Data Collection**	**QTRT1**	**QTRT1·Queuine**
PDB-ID	6H42	6H45
X-ray source	Synchrotron (PETRA III, P14)	Synchrotron (PETRA III, P14)
Detector	Dectris Eiger	Dectris Eiger
Wavelength (Å)	0.97626	0.97626
Space group	C2	C2
Cell dimensions		
a, b, c (Å)	127.70, 157.82, 47.77	127.50, 158.86, 47.62
α, β, γ (°)	90.00, 108.46, 90.00	90.00, 108.20, 90.00
Resolution (Å)	48.25–2.45 (2.55–2.45)	48.16–2.40 (2.50–2.40)
R_meas_ (%)	10.6 (111.2)	5.8 (59.0)
I/σ(I)	10.3 (1.7)	17.3 (2.3)
CC_1/2_	99.6 (59.9)	99.9 (84.7)
Completeness (%)	99.4 (99.5)	93.1 (67.6)
Redundancy	4.5 (4.5)	3.3 (2.7)
**Refinement**		
Resolution (Å)	48.25–2.45	48.16–2.40
No. of reflections	32849	32702
R_work_ (%)	20.24	20.13
R_free_ (%)	23.95	23.96
No. of atoms		
Protein	6033	6010
Water	99	103
Ligand	-	40
Ions	15	13
B-factors (Å^2^)		
Protein	67.3	58.0
Ligand	-	73.7
Water and other small molecules	61.7	71.7
r.m.s. deviations		
Bond lengths (Å)	0.0037	0.0074
Bond angles (°)	0.643	0.814
MolProbity analysis		
Ramachandran favored (%)	96.5	96.6
Ramachandran outliers (%)	0.0	0.0
Rotamer outliers (%)	1.37	1.99
Clash score	4.96	5.35

## Appendix B

Sequence alignment of a bacterial TGT and eukaryotic TGT subunits. Sequences of the *Z.mobilis* TGT and mouse QTRT2 have been aligned to the sequence of human QTRT1 (UniProtKB entries P28720 B8ZXI1 and Q9BXR0) using T-Coffee.

## References

[1] Boccaletto P., Machnicka M.A., Purta E., Piątkowski P., Bagiński B., Wirecki T.K., de Crécy-Lagard V., Ross R., Limbach P.A., Kotter A. (2018). MODOMICS: A database of RNA modification pathways. 2017 update. Nucleic Acids Res..

[2] Sokołowski M., Klassen R., Bruch A., Schaffrath R., Glatt S. (2018). Cooperativity between different tRNA modifications and their modification pathways. Biochim. Biophys. Acta BBA Gene Regul. Mech..

[3] Nachtergaele S., He C. (2017). The emerging biology of RNA post-transcriptional modifications. RNA Biol..

[4] Lorenz C., Lünse C.E., Mörl M. (2017). tRNA Modifications: Impact on structure and thermal adaptation. Biomolecules.

[5] Tuorto F., Herbst F., Alerasool N., Bender S., Popp O., Federico G., Reitter S., Liebers R., Stoecklin G., Gröne H.-J. (2015). The tRNA methyltransferase Dnmt2 is required for accurate polypeptide synthesis during haematopoiesis. EMBO J..

[6] Harada F., Nishimura S. (1972). Possible anticodon sequences of tRNA His, tRNA Asm, and tRNA Asp from *Escherichia coli* B. Universal presence of nucleoside Q in the first postion of the anticondons of these transfer ribonucleic acids. Biochemistry.

[7] Phillips G., Yacoubi B.E., Lyons B., Alvarez S., Iwata-Reuyl D., de Crécy-Lagard V. (2008). Biosynthesis of 7-Deazaguanosine-Modified tRNA Nucleosides: A New Role for GTP Cyclohydrolase I. J. Bacteriol..

[8] McCarty R.M., Somogyi Á., Bandarian V. (2009). *Escherichia coli* QueD Is a 6-Carboxy-5,6,7,8-Tetrahydropterin Synthase. Biochemistry.

[9] McCarty R.M., Somogyi Á., Lin G., Jacobsen N.E., Bandarian V. (2009). The deazapurine biosynthetic pathway revealed: In vitro enzymatic synthesis of PreQ0 from Guanosine 5′-Triphosphate in four steps. Biochemistry.

[10] Dowling D.P., Bruender N.A., Young A.P., McCarty R.M., Bandarian V., Drennan C.L. (2014). Radical SAM enzyme QueE defines a new minimal core fold and metal-dependent mechanism. Nat. Chem. Biol..

[11] Lanen S.G.V., Reader J.S., Swairjo M.A., de Crécy-Lagard V., Lee B., Iwata-Reuyl D. (2005). From cyclohydrolase to oxidoreductase: Discovery of nitrile reductase activity in a common fold. Proc. Natl. Acad. Sci. USA.

[12] Lee B.W.K., Van Lanen S.G., Iwata-Reuyl D. (2007). Mechanistic Studies of *Bacillus subtilis* QueF, the nitrile oxidoreductase involved in queuosine biosynthesis. Biochemistry.

[13] Chikwana V.M., Stec B., Lee B.W.K., de Crécy-Lagard V., Iwata-Reuyl D., Swairjo M.A. (2012). Structural basis of biological nitrile reduction. J. Biol. Chem..

[14] Goodenough-Lashua D.M., Garcia G.A. (2003). tRNA-Guanine Transglycosylase from *E. coli*: A Ping-Pong kinetic mechanism is consistent with nucleophilic catalysis. Bioorg. Chem..

[15] Xie W., Liu X., Huang R.H. (2003). Chemical trapping and crystal structure of a catalytic tRNA guanine transglycosylase covalent intermediate. Nat. Struct. Mol. Biol..

[16] Romier C., Reuter K., Suck D., Ficner R. (1996). Crystal structure of tRNA-guanine transglycosylase: RNA modification by base exchange. EMBO J..

[17] Romier C., Meyer J.E., Suck D. (1997). Slight sequence variations of a common fold explain the substrate specificities of tRNA-guanine transglycosylases from the three kingdoms. FEBS Lett..

[18] Ishitani R., Nureki O., Fukai S., Kijimoto T., Nameki N., Watanabe M., Kondo H., Sekine M., Okada N., Nishimura S. (2002). Crystal structure of archaeosine tRNA-guanine transglycosylase. J. Mol. Biol..

[19] Watanabe M., Nameki N., Matsuo-Takasaki M., Nishimura S., Okada N. (2001). tRNA Recognition of tRNA-guanine transglycosylase from a hyperthermophilic archaeon, *Pyrococcus horikoshii*. J. Biol. Chem..

[20] Ishitani R., Nureki O., Nameki N., Okada N., Nishimura S., Yokoyama S. (2003). Alternative tertiary structure of tRNA for recognition by a posttranscriptional modification enzyme. Cell.

[21] Romier C., Reuter K., Suck D., Ficner R. (1996). Mutagenesis and crystallographic studies of *Zymomonas mobilis* tRNA-guanine transglycosylase reveal aspartate 102 as the active site nucleophile. Biochemistry.

[22] Garcia G.A., Chervin S.M., Kittendorf J.D. (2009). Identification of the rate-determining step of tRNA-guanine transglycosylase from *Escherichia coli*. Biochemistry.

[23] Durand J.M., Okada N., Tobe T., Watarai M., Fukuda I., Suzuki T., Nakata N., Komatsu K., Yoshikawa M., Sasakawa C. (1994). vacC, a virulence-associated chromosomal locus of *Shigella flexneri*, is homologous to tgt, a gene encoding tRNA-guanine transglycosylase (Tgt) of *Escherichia coli* K-12. J. Bacteriol..

[24] Durand J.M.B., Dagberg B., Uhlin B.E., Björk G.R. (2000). Transfer RNA modification, temperature and DNA superhelicity have a common target in the regulatory network of the virulence of *Shigella flexneri*: The expression of the virF gene. Mol. Microbiol..

[25] Hurt J.K., Olgen S., Garcia G.A. (2007). Site-specific modification of *Shigella flexneri virF* mRNA by tRNA-guanine transglycosylase in vitro. Nucleic Acids Res..

[26] Barandun L.J., Immekus F., Kohler P.C., Ritschel T., Heine A., Orlando P., Klebe G., Diederich F. (2013). High-affinity inhibitors of *Zymomonas mobilis* tRNA-guanine transglycosylase through convergent optimization. Acta Crystallogr. D Biol. Crystallogr..

[27] Immekus F., Barandun L.J., Betz M., Debaene F., Petiot S., Sanglier-Cianferani S., Reuter K., Diederich F., Klebe G. (2013). Launching spiking ligands into a protein-protein interface: A promising strategy to destabilize and break interface formation in a tRNA modifying enzyme. ACS Chem. Biol..

[28] Jeltsch A., Ehrenhofer-Murray A., Jurkowski T.P., Lyko F., Reuter G., Ankri S., Nellen W., Schaefer M., Helm M. (2017). Mechanism and biological role of Dnmt2 in Nucleic Acid Methylation. RNA Biol..

[29] Reyniers J.P., Pleasants J.R., Wostmann B.S., Katze J.R., Farkas W.R. (1981). Administration of exogenous queuine is essential for the biosynthesis of the queuosine-containing transfer RNAs in the mouse. J. Biol. Chem..

[30] Katze J.R., Gündüz U., Smith D.L., Cheng C.S., McCloskey J.A. (1984). Evidence that the nucleic acid base queuine is incorporated intact into tRNA by animal cells. Biochemistry.

[31] Chen Y.C., Kelly V.P., Stachura S.V., Garcia G.A. (2010). Characterization of the human tRNA-guanine transglycosylase: Confirmation of the heterodimeric subunit structure. RNA.

[32] Behrens C., Biela I., Petiot-Bécard S., Botzanowski T., Cianférani S., Sager C.P., Klebe G., Heine A., Reuter K. (2018). Homodimer architecture of QTRT2, the noncatalytic subunit of the eukaryotic tRNA-Guanine Transglycosylase. Biochemistry.

[33] Baranowski W., Dirheimer G., Jakowicki J.A., Keith G. (1994). Deficiency of queuine, a highly modified purine base, in transfer RNAs from primary and metastatic ovarian malignant tumors in women. Cancer Res..

[34] Huang B.-S., Wu R.-T., Chien K.-Y. (1992). Relationship of the queuine content of transfer ribonucleic acids to histopathological grading and survival in human lung cancer. Cancer Res..

[35] Dirheimer G., Baranowski W., Keith G. (1995). Variations in tRNA modifications, particularly of their queuine content in higher eukaryotes. Its relation to malignancy grading. Biochimie.

[36] Rakovich T., Boland C., Bernstein I., Chikwana V.M., Iwata-Reuyl D., Kelly V.P. (2011). Queuosine deficiency in eukaryotes compromises tyrosine production through increased tetrahydrobiopterin oxidation. J. Biol. Chem..

[37] Reisser T., Langgut W., Kersten H. (1994). The nutrient factor queuine protects HeLa cells from hypoxic stress and improves metabolic adaptation to oxygen availability. Eur. J. Biochem..

[38] Johannsson S., Neumann P., Wulf A., Welp L.M., Gerber H.-D., Krull M., Diederichsen U., Urlaub H., Ficner R. (2018). Structural insights into the stimulation of *S. pombe* Dnmt2 catalytic efficiency by the tRNA nucleoside queuosine. Sci. Rep..

[39] Müller M., Hartmann M., Schuster I., Bender S., Thüring K.L., Helm M., Katze J.R., Nellen W., Lyko F., Ehrenhofer-Murray A.E. (2015). Dynamic modulation of Dnmt2-dependent tRNA methylation by the micronutrient queuine. Nucleic Acids Res..

[40] Kabsch W. (2010). XDS. Acta Crystallogr. D Biol. Crystallogr..

[41] McCoy A.J., Grosse-Kunstleve R.W., Adams P.D., Winn M.D., Storoni L.C., Read R.J. (2007). Phaser crystallographic software. J. Appl. Crystallogr..

[42] Winn M.D., Ballard C.C., Cowtan K.D., Dodson E.J., Emsley P., Evans P.R., Keegan R.M., Krissinel E.B., Leslie A.G.W., McCoy A. (2011). Overview of the CCP4 suite and current developments. Acta Crystallogr. D Biol. Crystallogr..

[43] Leaver-Fay A., Tyka M., Lewis S.M., Lange O.F., Thompson J., Jacak R., Kaufman K., Renfrew P.D., Smith C.A., Sheffler W. (2011). ROSETTA3: An object-oriented software suite for the simulation and design of macromolecules. Methods Enzymol..

[44] Adams P.D., Afonine P.V., Bunkóczi G., Chen V.B., Davis I.W., Echols N., Headd J.J., Hung L.-W., Kapral G.J., Grosse-Kunstleve R.W. (2010). PHENIX: A comprehensive Python-based system for macromolecular structure solution. Acta Crystallogr. D Biol. Crystallogr..

[45] Emsley P., Lohkamp B., Scott W.G., Cowtan K. (2010). Features and development of Coot. Acta Crystallogr. D Biol. Crystallogr..

[46] Brenk R., Stubbs M.T., Heine A., Reuter K., Klebe G. (2003). Flexible adaptations in the structure of the tRNA-modifying enzyme tRNA-Guanine transglycosylase and their implications for substrate selectivity, reaction mechanism and structure-based drug design. ChemBioChem.

[47] Biela I., Tidten-Luksch N., Immekus F., Glinca S., Nguyen T.X.P., Gerber H.-D., Heine A., Klebe G., Reuter K. (2013). Investigation of specificity determinants in bacterial tRNA-Guanine transglycosylase reveals queuine, the substrate of its eucaryotic counterpart, as inhibitor. PLoS ONE.

[48] Krissinel E., Henrick K. (2007). Inference of macromolecular assemblies from crystalline state. J. Mol. Biol..

[49] Goll M.G., Kirpekar F., Maggert K.A., Yoder J.A., Hsieh C.-L., Zhang X., Golic K.G., Jacobsen S.E., Bestor T.H. (2006). Methylation of tRNA^Asp^ by the DNA Methyltransferase Homolog Dnmt2. Science.

[50] Kasai H., Nakanishi K., Macfarlane R.D., Torgerson D.F., Ohashi Z., McCloskey J.A., Gross H.J., Nishimura S. (1976). The structure of Q* nucleoside isolated from rabbit liver transfer ribonucleic acid. J. Am. Chem. Soc..

[51] Nishimura S., Cohn W.E. (1983). Structure, biosynthesis, and function of queuosine in transfer RNA. Progress in Nucleic Acid Research and Molecular Biology.

